# Delayed Diagnosis of an Ipsilateral Patellar Fracture in an Open Segmental Tibial Fracture Managed With Ilizarov External Fixation: A Case Report

**DOI:** 10.7759/cureus.106591

**Published:** 2026-04-07

**Authors:** Md Sulaiman

**Affiliations:** 1 Orthopedic Surgery, Trauma General Hospital and Diagnostic Center, Brahmanbaria, BGD

**Keywords:** delayed diagnosis, ilizarov external fixation, open tibial fracture, patella fracture, segmental tibial fracture

## Abstract

Open segmental tibial fractures are challenging injuries because of their high-energy mechanism, associated soft tissue compromise, and increased risk of infection and delayed union. Ilizarov circular external fixation provides stable fixation while preserving fracture biology and allowing early rehabilitation. This report describes the case of a 45-year-old male who sustained a Gustilo-Anderson type I open segmental comminuted fracture of the right tibia with an associated ipsilateral mid-shaft fibula fracture. Definitive management was performed with wound debridement and Ilizarov circular external fixation. During follow-up, an ipsilateral patellar fracture that had not been identified at initial presentation was diagnosed radiographically and subsequently treated with modified tension band wiring. Follow-up radiographs demonstrated maintained alignment, progressive callus formation, and progression toward union, while postoperative patella radiographs showed satisfactory fixation. This case highlights the effectiveness of Ilizarov circular external fixation in the management of open segmental tibial fractures and emphasizes the importance of careful evaluation of adjacent joints to avoid delayed diagnosis of associated injuries.

## Introduction

Tibial shaft fractures are among the most common long bone injuries and are frequently the result of high-energy trauma, particularly in road traffic accidents. Open and segmental tibial fractures are associated with significant soft tissue damage, an increased risk of infection, and challenges in achieving stable fixation and timely union. External fixation, including Ilizarov-based techniques, remains an effective option for complex tibial diaphyseal and segmental fractures [1-3].

However, high-energy mechanisms can also result in concomitant peri-knee injuries that may be overlooked during the initial assessment. Failure to identify associated injuries can lead to delayed diagnosis despite structured trauma evaluation, and missed injuries remain a recognized problem in severely injured patients [4,5].

This report describes a case of delayed diagnosis of an ipsilateral patellar fracture associated with an open segmental tibial shaft fracture managed with Ilizarov external fixation. This case highlights the importance of a systematic secondary survey and careful evaluation of the knee joint in patients presenting with high-energy tibial fractures.

## Case presentation

**Figure 1:**
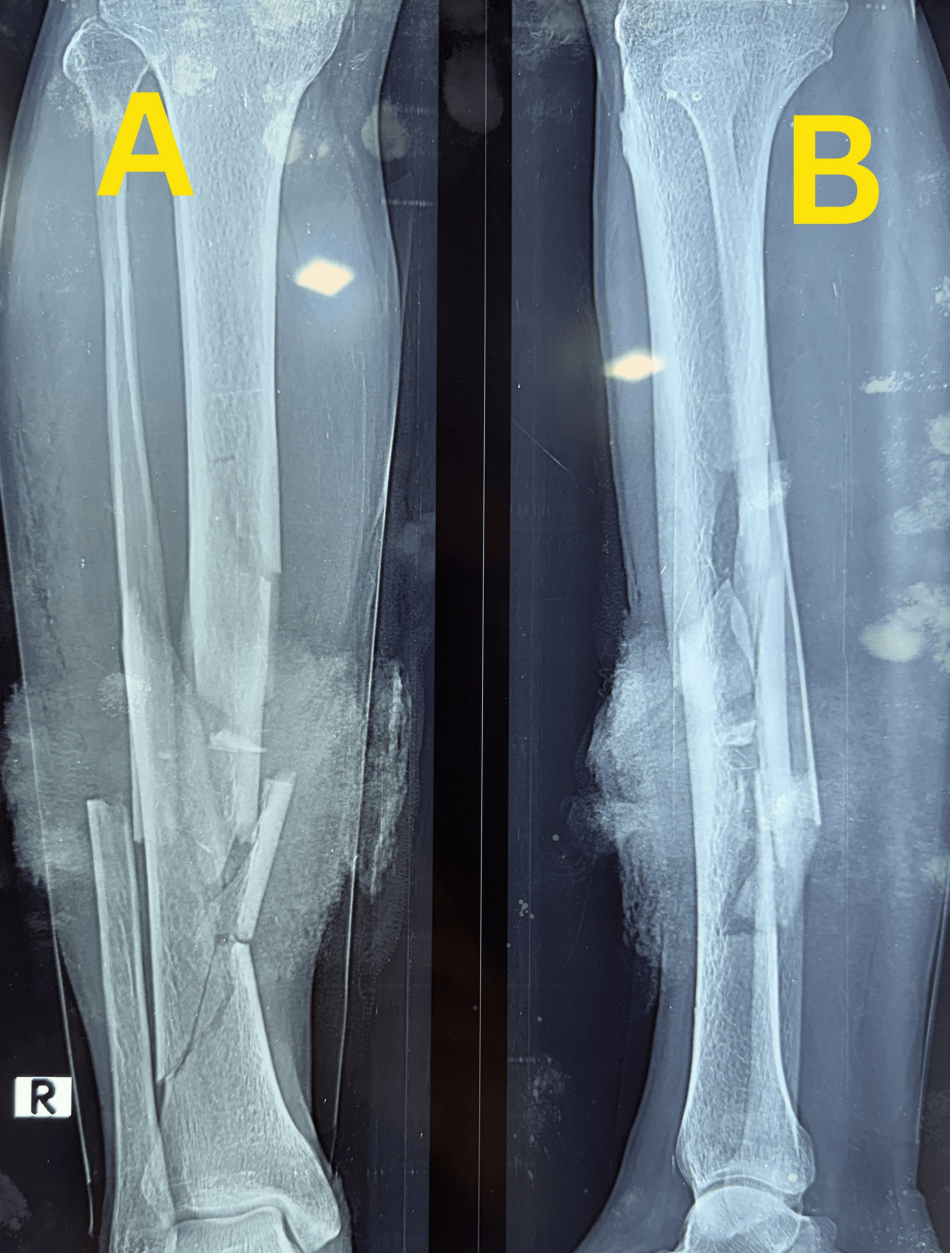
Preoperative radiographs of the right leg Preoperative anteroposterior (A) and lateral (B) radiographs of the right leg obtained on 14 November 2025, demonstrating a segmental comminuted fracture of the tibia with an associated ipsilateral mid-shaft fibula fracture.

A 45-year-old male sustained a high-energy injury to the right leg following a road traffic accident on 14 November 2025 and was treated at a tertiary care hospital. Initial clinical and radiographic evaluation revealed a Gustilo-Anderson type I open segmental comminuted fracture of the right tibia with an associated ipsilateral mid-shaft fibula fracture (Figure 1).

The open wound measured approximately 2 cm. Neurovascular examination of the affected limb was normal. Initial assessment was directed primarily toward the open tibial injury, and detailed knee examination was limited by pain and soft tissue swelling. Dedicated knee radiographs were not obtained at presentation.

At presentation, intravenous antibiotic prophylaxis was started promptly, tetanus prophylaxis was given, and the wound was covered with a sterile dressing with temporary immobilization of the limb. Because the wound was small and classified as Gustilo-Anderson type I, definitive fixation was performed on 16 November 2025 after initial stabilization, preoperative optimization, and surgical planning.

**Figure 2:**
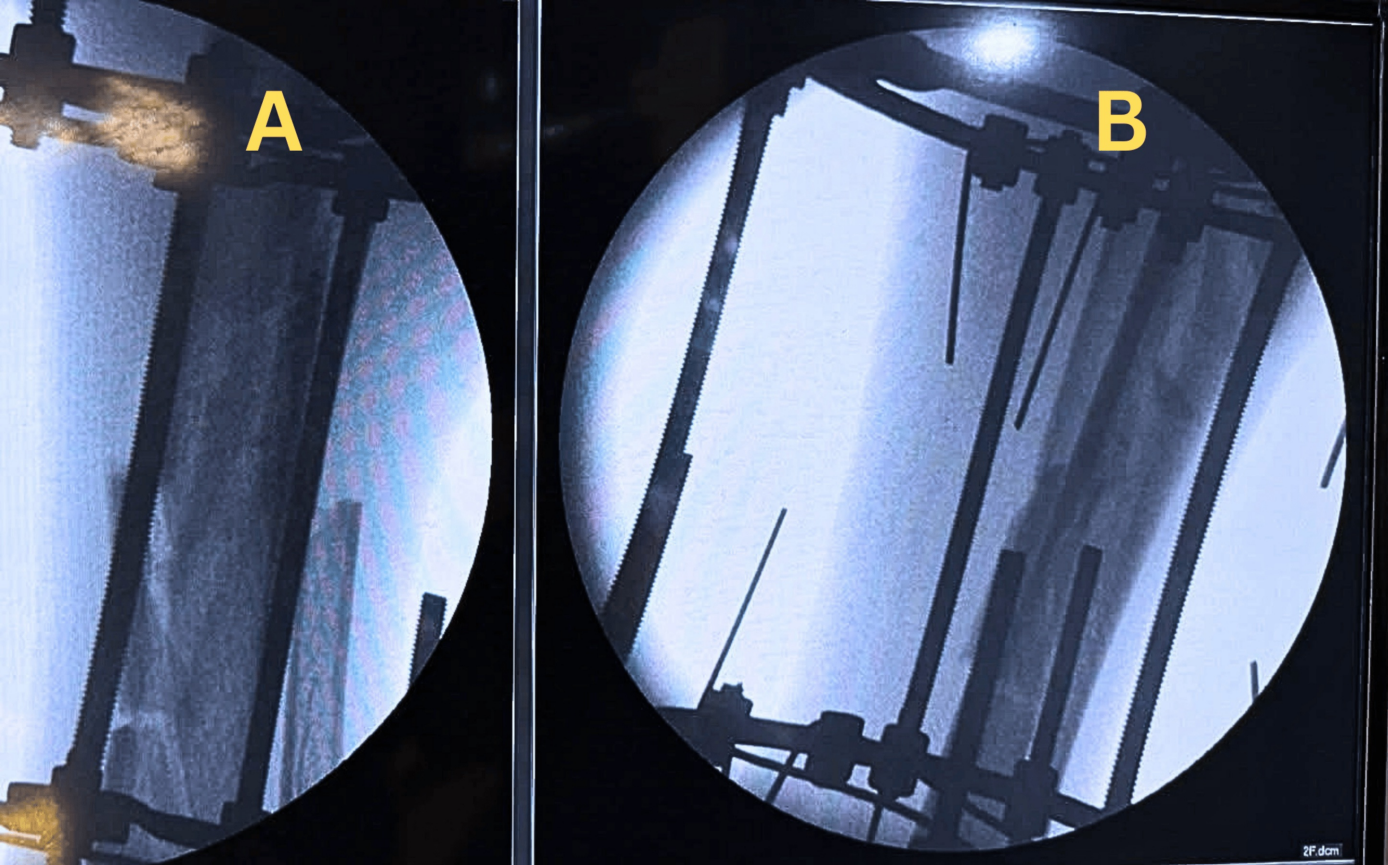
Intraoperative fluoroscopic image after Ilizarov fixation Intraoperative fluoroscopic images after application of the Ilizarov circular external fixator showing reduction and stabilization of the segmental tibial fracture in two orthogonal views: (A) anteroposterior view and (B) lateral view.

On 16 November 2025, the patient underwent thorough wound irrigation and debridement followed by stabilization of the tibial fracture with an Ilizarov circular external fixator. Because the wound was small and clean, primary closure was performed. The construct consisted of a four-ring frame using eight 1.8-mm tensioned K-wires, without half-pins, providing stable fixation of both tibial fracture segments while minimizing additional soft tissue insult. Intraoperative fluoroscopic imaging (C-arm) was used to confirm fracture reduction, wire placement, frame position, and satisfactory alignment of both tibial fracture segments after Ilizarov application (Figure 2).

The patient was subsequently mobilized with weight-bearing as tolerated, with gradual progression according to pain tolerance and clinical assessment. Rehabilitation during follow-up included pin-site care, knee and ankle range-of-motion exercises, and progressive ambulation under guidance from the treating team.

**Figure 3:**
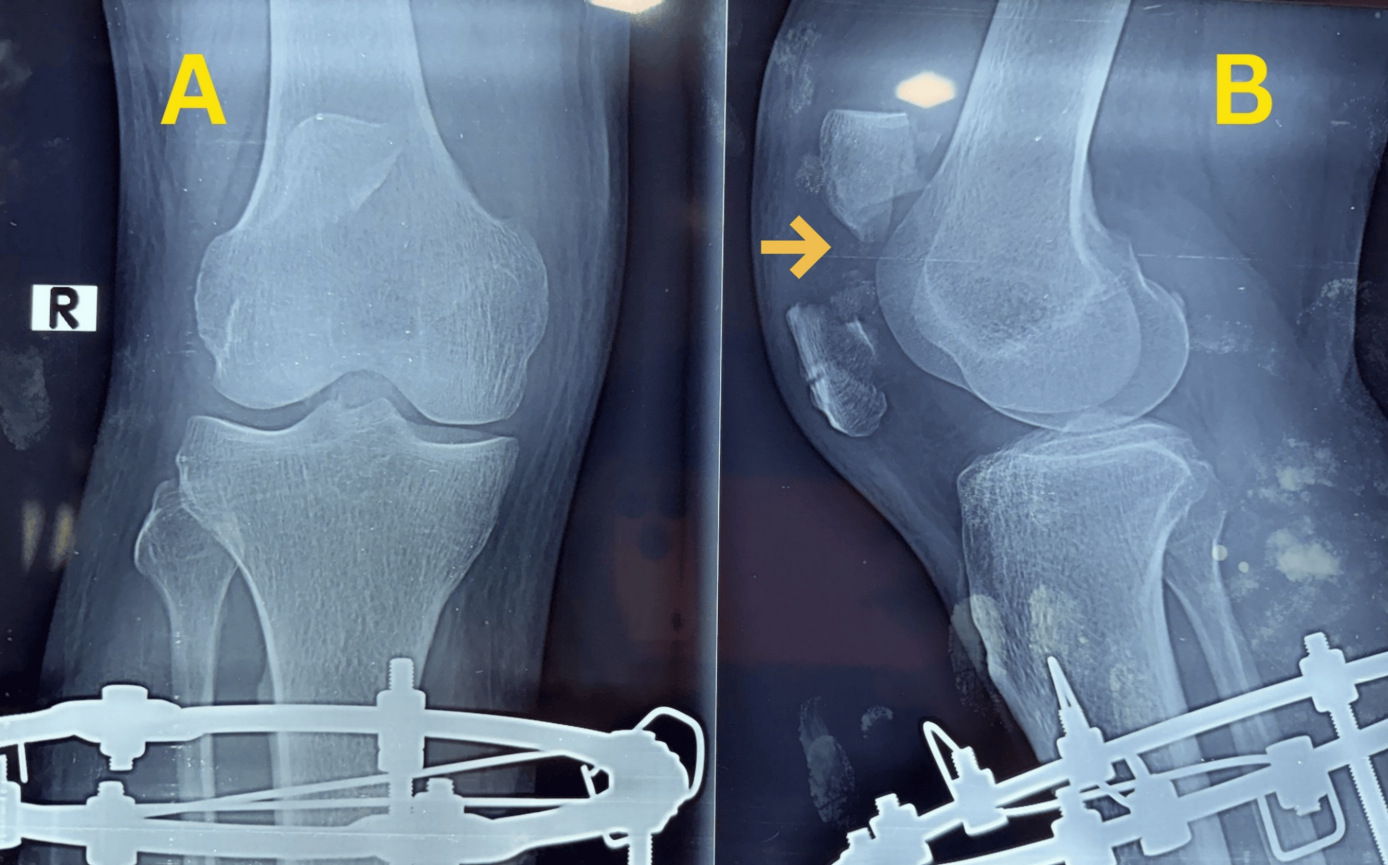
Radiograph showing delayed diagnosis of patellar fracture Knee radiographs obtained after surgery demonstrating the associated patellar fracture: (A) anteroposterior view and (B) lateral view. The arrow indicates the patellar fracture site.

During follow-up, the patient developed persistent anterior knee pain, which prompted further reassessment. A radiograph obtained on 25 November 2025 demonstrated a previously unrecognized ipsilateral transverse mid-patellar fracture with mild comminution (Figure 3).

The diagnosis was made during reassessment, highlighting the potential for associated peri-knee injuries to be overlooked in the setting of severe open tibial trauma. Exact displacement measurement and formal documentation of extensor mechanism status were not available on retrospective review.

**Figure 4:**
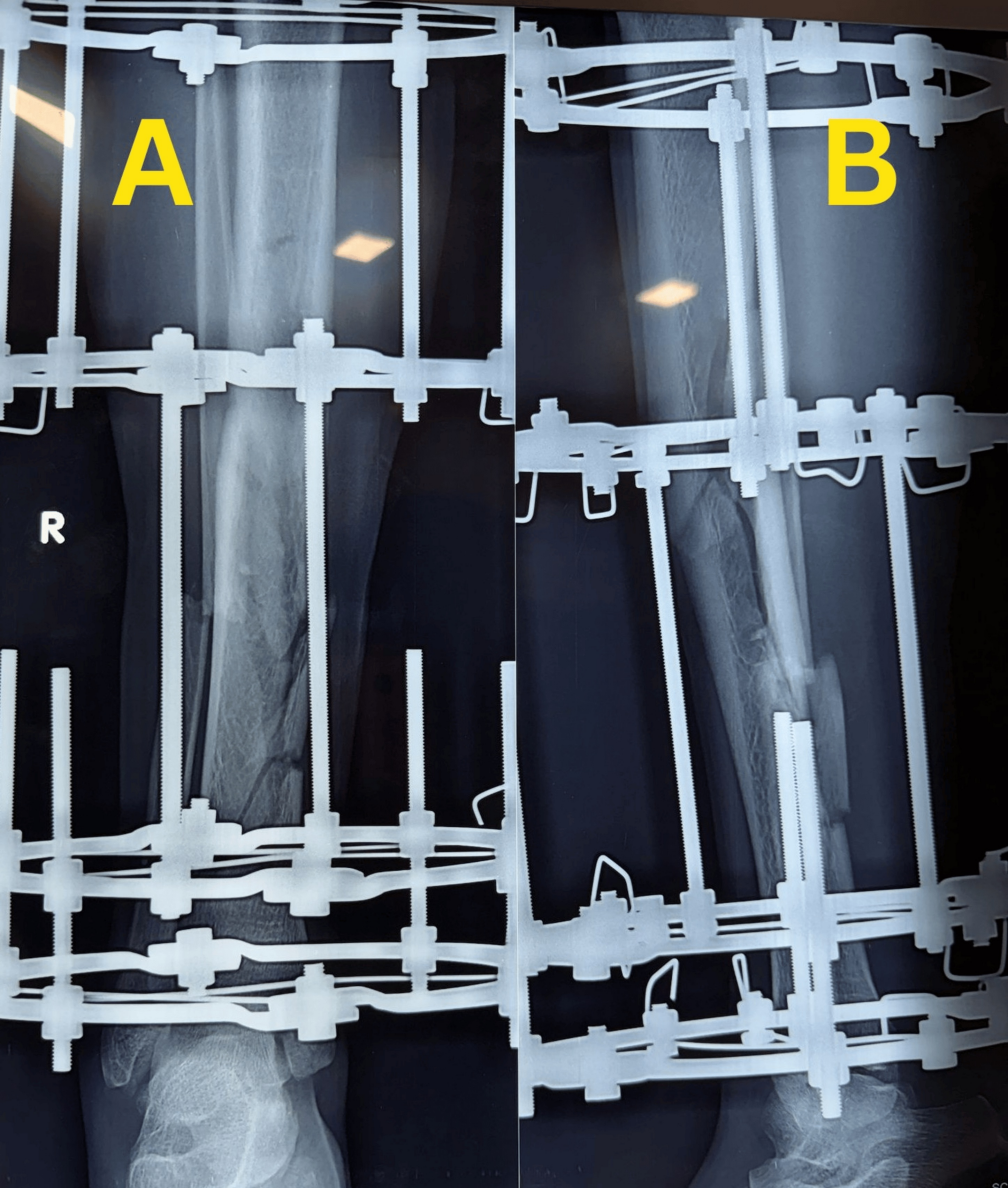
Follow-up radiographs of the right tibia Follow-up radiographs obtained on 23 December 2025 demonstrating maintained alignment of the tibial fracture with early callus formation: anteroposterior view (A) and lateral view (B).

Subsequent follow-up radiographs obtained on 23 December 2025 demonstrated maintained alignment of the tibial fracture with early callus formation (Figure 4).

**Figure 5:**
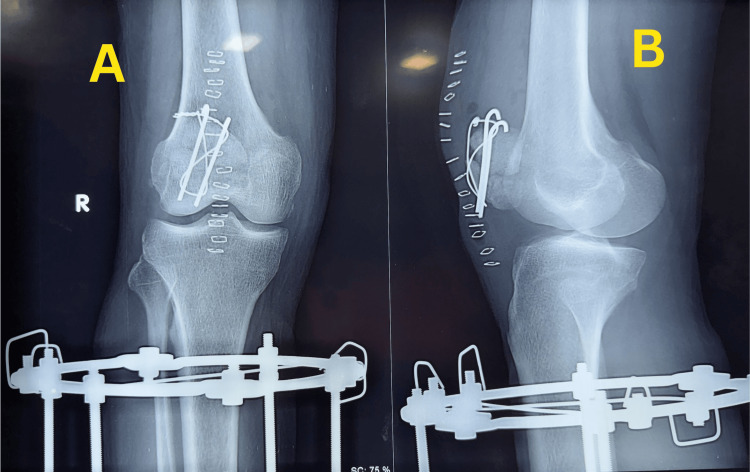
Postoperative radiographs after patellar fracture fixation Postoperative radiographs obtained on 23 December 2025, demonstrating fixation of the ipsilateral patellar fracture using modified tension band wiring following surgery performed on 21 December 2025: anteroposterior view (A) and lateral view (B).

Because of the delayed diagnosis of the patellar fracture, the patient underwent operative fixation with modified tension band wiring on 21 December 2025. The interval between diagnosis and surgical fixation was due to the patient’s preference to delay surgery. Postoperative radiographs obtained on 23 December 2025 confirmed satisfactory fixation of the patellar fracture (Figure 5).

**Figure 6:**
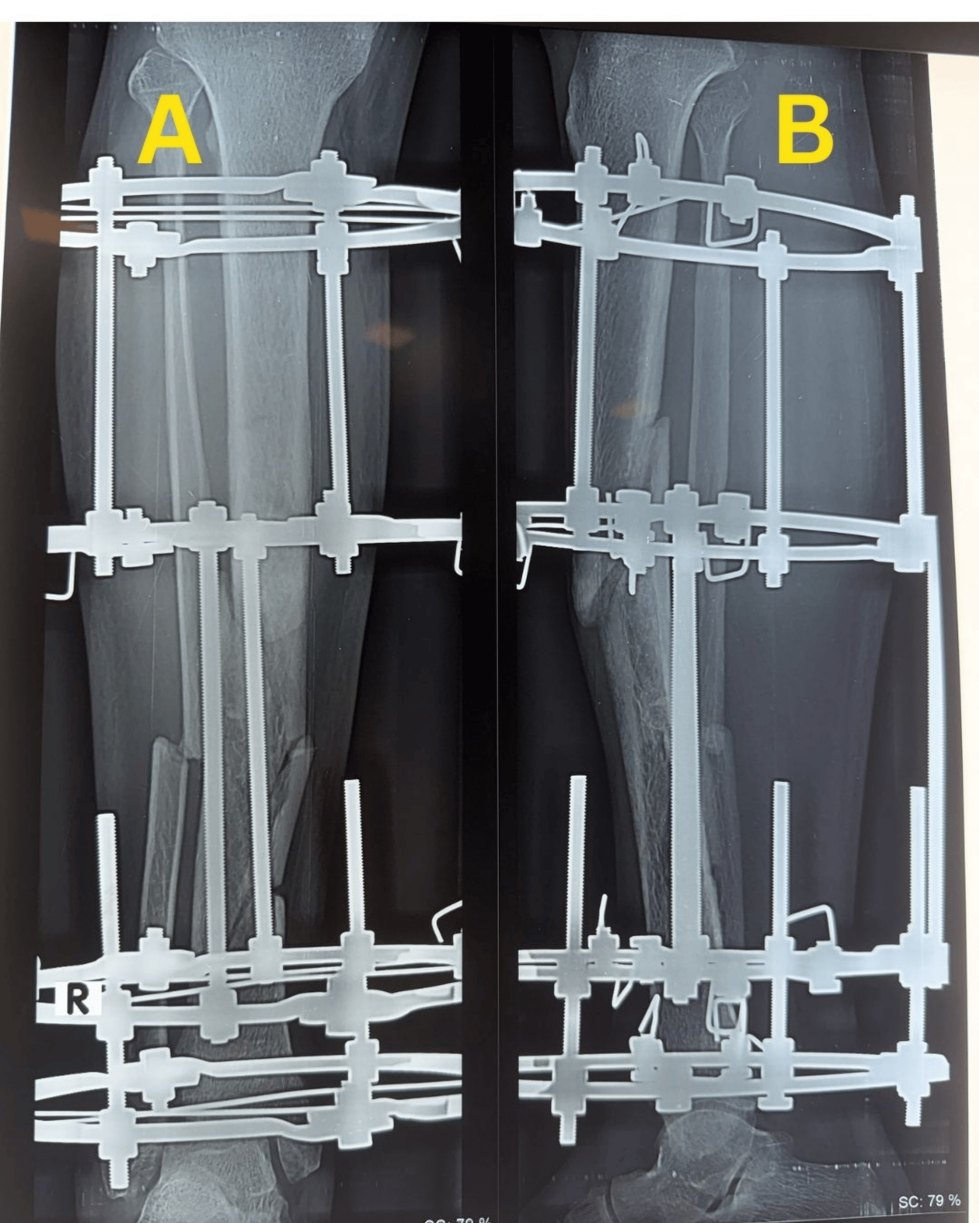
Follow-up radiographs showing progressive fracture healing Follow-up radiographs obtained on 18 February 2026 showing progressive callus formation and ongoing fracture healing: anteroposterior view (A) and lateral view (B).

The surgical wound healed satisfactorily without evidence of infection, and serial follow-up showed uncomplicated soft tissue recovery. Further follow-up radiographs obtained on 18 February 2026 showed progressive callus formation and ongoing fracture healing (Figure 6).

**Figure 7:**
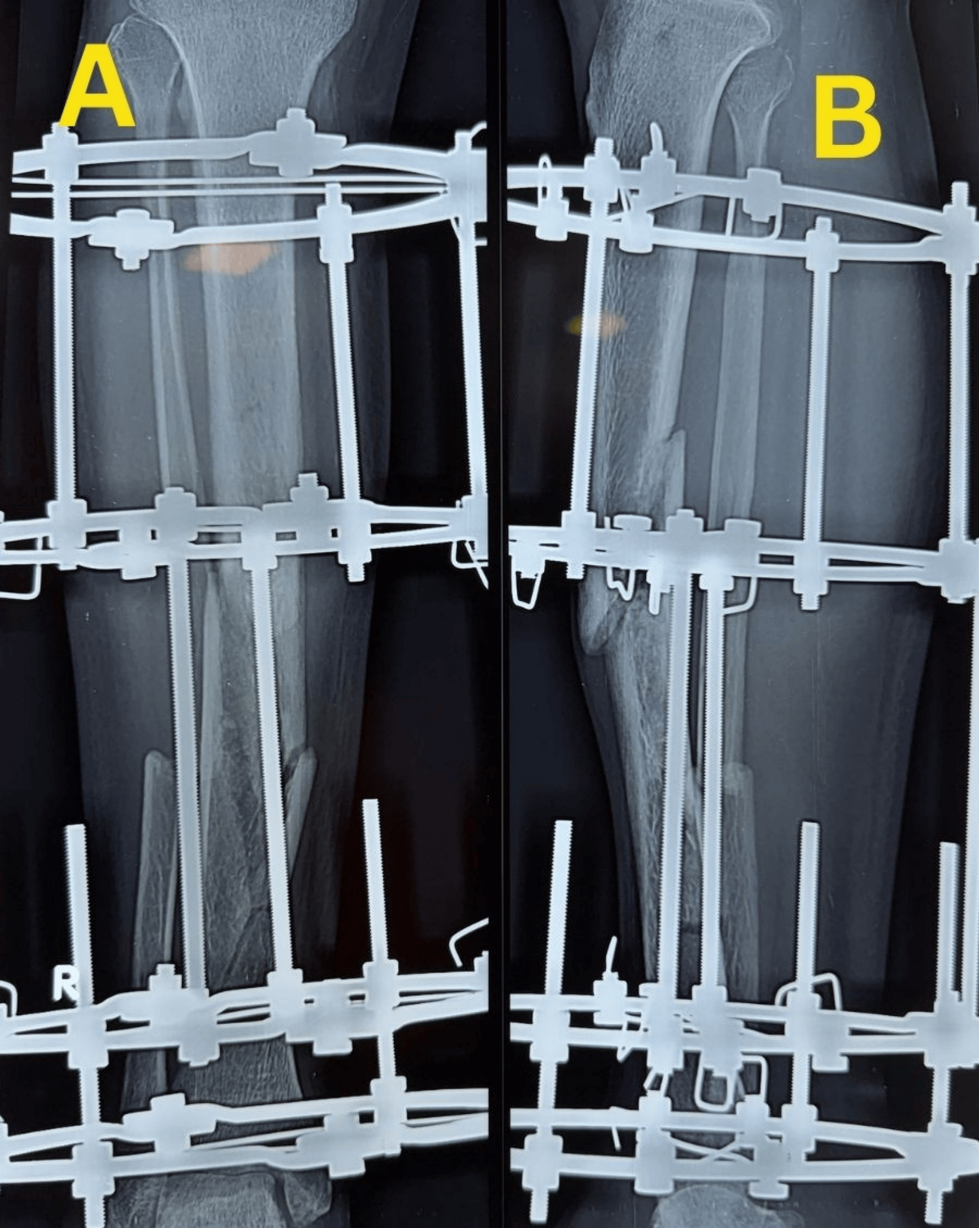
Follow-up radiographs showing progressive consolidation Follow-up radiographs obtained on 11 March 2026 demonstrating progressive consolidation and progression toward union: anteroposterior view (A) and lateral view (B).

Radiographs obtained on 11 March 2026 demonstrated progressive consolidation and progression toward union (Figure 7).

**Figure 8:**
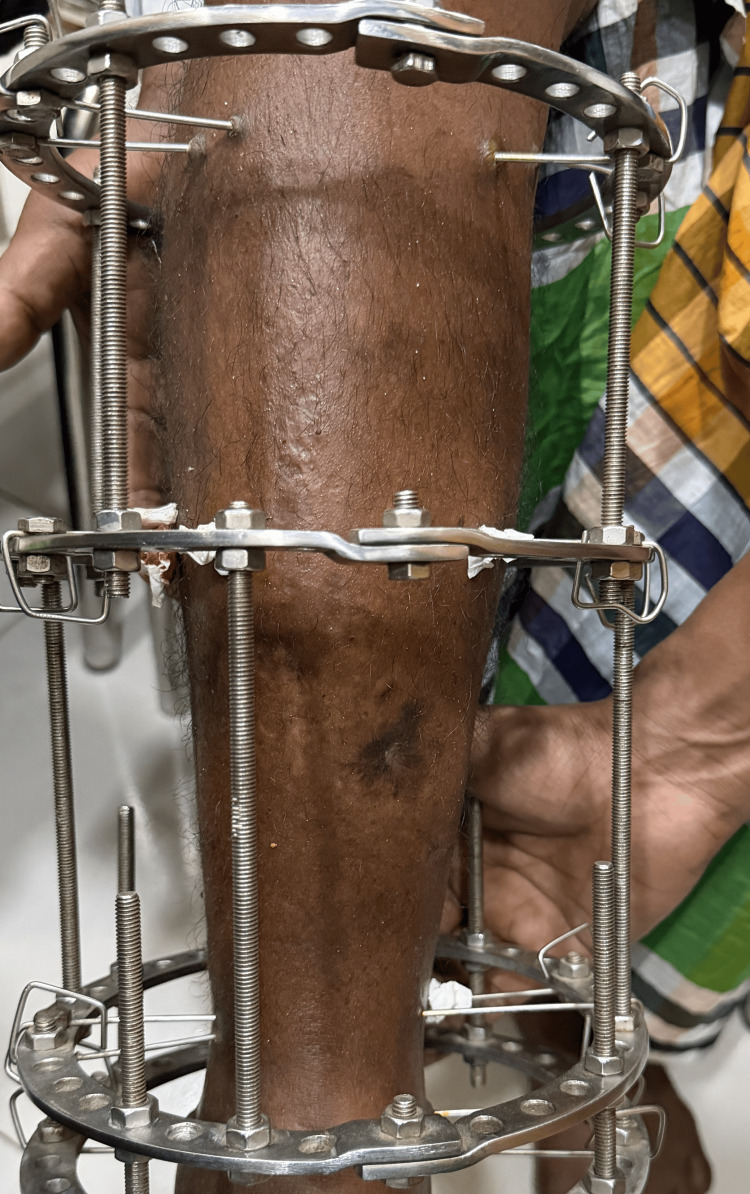
Clinical photograph showing Ilizarov external fixator in situ Clinical photograph of the right leg showing the Ilizarov circular external fixator in situ.

During follow-up, the patient remained ambulatory, although knee flexion was restricted by the Ilizarov rings. A clinical photograph demonstrated the Ilizarov circular external fixator in situ on the right leg (Figure 8).

At the last documented follow-up, the frame remained in situ, and continued serial clinical and radiographic follow-up was planned until sufficient consolidation was achieved for frame removal.

Overall, the patient showed satisfactory radiological progression of tibial fracture healing after Ilizarov fixation, and the patellar fracture was successfully managed despite delayed diagnosis.

## Discussion

High-energy tibial fractures are frequently associated with extensive soft tissue injury, and the severity of the primary lesion can easily divert attention from associated injuries around the knee. In open segmental tibial fractures, the immediate priorities usually include wound assessment, contamination control, fracture stabilization, and preservation of limb viability. In this setting, associated peri-knee injuries may be overlooked, particularly when pain, swelling, and the overall trauma burden limit a complete examination. Missed and delayed diagnoses remain recognized problems in severely injured trauma patients despite structured emergency assessment protocols [4,5].

The present case illustrates this challenge clearly. Initial management was appropriately focused on the open segmental tibial fracture, while the associated patellar fracture was not identified at presentation. In severe open tibial trauma, formal assessment of the extensor mechanism may be difficult or unreliable at the initial stage because pain, swelling, and the urgency of treating the limb-threatening injury can limit a detailed knee examination. This highlights the importance of a structured secondary survey in similar high-energy injuries. In practical terms, reassessment should include repeated clinical examination of the joints above and below the fracture, documentation of local tenderness and swelling, and, where feasible, documentation of extensor mechanism function, and a low threshold for dedicated radiographs when symptoms or mechanism raises suspicion of associated peri-articular injury. In this patient, persistent anterior knee pain during follow-up prompted further evaluation and led to the identification of the ipsilateral patellar fracture. A more comprehensive initial radiographic assessment might have facilitated earlier diagnosis, although the complexity of the primary injury understandably dominated the initial management. This sequence shows how delayed diagnosis may occur even in appropriately managed trauma settings and reinforces the need for vigilance beyond the primary injury [4,5].

Another important issue is the clinical impact of delayed recognition. Although the patellar fracture was diagnosed after initial tibial stabilization and fixed at a later stage, the patient still showed satisfactory radiological progression and acceptable overall recovery. This suggests that delayed diagnosis does not necessarily result in a poor outcome if the injury is subsequently recognized and managed appropriately. Nevertheless, such a delay may prolong symptoms, postpone targeted treatment, and complicate rehabilitation planning. When a peri-knee injury coexists with a circular external fixator, recovery may become more difficult because pain, stiffness, and mechanical restriction from the frame can all affect knee function. Earlier recognition of associated knee pathology would allow more coordinated treatment planning and more focused rehabilitation.

The tibial fracture itself was managed effectively with Ilizarov circular external fixation. Previous studies have shown that Ilizarov and other external fixation techniques can provide stable fixation for complex tibial diaphyseal and segmental fractures while preserving the biological environment needed for union [1-3,6]. This is particularly relevant in open injuries, where minimizing additional soft tissue insult is a major advantage. In the present case, the frame provided stable fixation across both tibial fracture segments and allowed progressive fracture healing. These features support the role of circular external fixation as a useful definitive treatment option in selected open segmental tibial fractures, particularly when soft tissue considerations make extensive internal fixation less desirable [1-3,6].

From an educational perspective, this case underscores the importance of a structured and repeated assessment strategy in similar trauma scenarios. When managing a high-energy open tibial fracture, clinicians should maintain a low threshold for careful knee examination and additional imaging if symptoms, mechanism of injury, or follow-up findings suggest associated peri-articular pathology. Missed injuries are not always the result of poor initial care; they often reflect the complexity of severe trauma itself. Even so, awareness of this risk and deliberate reassessment can reduce the risk of delayed diagnosis and improve overall management [4,5].

Overall, this case highlights two important lessons. First, high-energy open tibial fractures may coexist with peri-knee injuries that are not apparent during the initial evaluation. Second, Ilizarov external fixation remains a reliable option for stabilizing complex open tibial fractures. Still, associated injuries around the knee must be actively sought so that staged treatment and rehabilitation can be planned more effectively [1-6].

## Conclusions

High-energy open tibial fractures may be associated with concomitant peri-knee injuries that can be overlooked during the initial assessment. Highlighting the need for thorough and repeated clinical evaluation can reinforce the importance of their role in patient care, making them feel responsible for optimal outcomes. Early recognition and appropriate management of associated injuries are important for optimizing clinical and radiological results, even when diagnosis is delayed.

## References

[1] Oztürkmen Y, Karamehmetoğlu M, Karadeniz H, Azboy I, Caniklioğlu M (2009). Acute treatment of segmental tibial fractures with the Ilizarov method. Injury.

[2] Albushtra A, Mohsen AH, Alnozaili KA, Ahmed F, Aljobahi YM, Mohammed F, Badheeb M (2024). External fixation as a primary and definitive treatment for complex tibial diaphyseal fractures: an underutilized and efficacious approach. Orthop Res Rev.

[3] Beltsios M, Savvidou O, Kovanis J, Alexandropoulos P, Papagelopoulos P (2009). External fixation as a primary and definitive treatment for tibial diaphyseal fractures. Strategies Trauma Limb Reconstr.

[4] Ju E, Baek SY, Hong SS, Kim Y, Youn SH (2022). An analysis of missed injuries in patients with severe trauma. J Trauma Inj.

[5] Suda AJ, Baran K, Brunnemer S, Köck M, Obertacke U, Eschmann D (2022). Delayed diagnosed trauma in severely injured patients despite guidelines-oriented emergency room treatment: there is still a risk. Eur J Trauma Emerg Surg.

[6] Pasetto VR, Morais IH, Faria FF (2025). Epidemiological profile of patients with tibial fractures treated with the Ilizarov external fixator. Cureus.

